# Controlling the Surface Morphology of ZnO Nano-Thin Film Using the Spin Coating Technique

**DOI:** 10.3390/ma15176178

**Published:** 2022-09-05

**Authors:** I. A. Elsayed, Ahmed S. Afify

**Affiliations:** 1 Department of Physics, Faculty of Science, Damietta University, New Damietta 34517, Egypt; 2 Department of Basic Sciences, The Higher Institute for Engineering, Automotive Technology and Energy, New Heliopolis 11829, Egypt

**Keywords:** optoelectronic, solar cells, spin coating, thin films, ZnO

## Abstract

Zinc oxide (ZnO) thin films are significant in various electronic applications. This study introduced an efficient, simple, low cost and timesaving method to obtain an extended and uniform ZnO thin film with tunable surface morphology over the substrate using the spin coating technique. Different concentrations of zinc acetate dehydrate were used as precursor solutions mixed with polyvinyl alcohol as a binding polymer to obtain the film’s uniformity and to relieve thermal expansion that may cause a wrinkled surface. Synthesized films were characterized using X-ray diffraction (XRD), X-ray spectroscopy (XPS), scanning electron microscopy (SEM), atomic force microscopy (AFM) and ellipsometry. Findings show that the average size of ZnO particles was less than 50 nm in a uniform film over the whole substrate area regardless of the presence or absence of wrinkles. Additionally, this method was quite fast, attaining the desired viscosity in less than one hour in comparison with the time-consuming aging method, which requires approximately 24 h to achieve the required viscosity.

## 1. Introduction

Polymer solar cells based on bulk-heterojunction and perovskite solar cells are considered by many to be complementary solutions for global energy demand [1,2,3,4]. Polymer and perovskite solar cells’ conventional structures experience some weaknesses that researchers have been attempting to overcome using inverted structure polymer solar cells, in which charges are collected inversely [5,6,7,8,9,10]. In this inverted structure, a cathode buffer layer (CBL) is sandwiched between the transparent conducting electrode and the active layer. A CBL performs crucial roles such as achieving durability, efficiency, etc. [9,11,12]. Firstly, it isolates the direct contact between the acidic active layer and the transparent conduction electrode and stops electrode etching due to the acidity of the active layer [5,13]. Secondly, the CBL’s energy band structure helps extract electrons and block holes; it is also known as the electron selective layer (ESL) or electron transporting layer (ETL) [2,14]. Finally, it permits the usage of high work function metal, such as Ag, as a counter electrode, which is more resistive to severe environmental conditions. For these reasons, it is essential to understand and control all aspects of the CBL layer, including its structural, optical and electrical properties. Zinc oxide (ZnO) is one of the most widely used CBL materials [9,10,12]. ZnO is an n-type material with a direct wide band gap of 3.30 eV [15] which makes it suitable to be widely used in optoelectronics applications such as gas sensors, diode lasers and polymer solar cells [12,16,17,18]. A ZnO layer can be deposited using many different techniques, including molecular beam epitaxy (MBE), chemical vapor deposition (CVD), RF sputtering, pulsed laser deposition (PLD), sprays pyrolysis, etc. [19,20,21,22,23]. Among these methods, spin coating is considered one of the simplest, low cost and timesaving techniques [24,25,26,27,28]. Spin coating ZnO encounters two major challenges, the uniformity and morphology of the obtained films [9,29], which impact polymer and perovskite solar cell performance [9,10,27,28,29]. To achieve a uniform ZnO film, the viscosity of the precursor solution must be adjusted. Nevertheless, to manufacture a uniform film, thermal expansion of the substrate during heat treatment acts as a negative support, which builds a ripple or wrinkle on the entire film surface; these wrinkles increase the surface area of the film. Although many researchers agree that wrinkles in the film of ZnO gas sensors increase their performance, there are debates regarding the effect of ZnO film wrinkles on solar cells’ performance [2,17,30,31,32,33,34].

The polymer assist deposition (PAD) method was used in this study; we added polymeric binding material which is polyvinyl alcohol (PVA) to the precursor solution [34,35,36,37,38] to aid the uniformy distribution of the zinc acetate, and to speed up the solution processing time by immediately providing the required viscosity (instead of aging). In addition, the PVA functioned as a spacer in the xerogel film, which can prevent the stress expected during the substrate’s thermal expansion [35,36,37,38,39].

The aim of this research was to introduce a reliable and reproducible procedure that yields extended uniform ZnO film, with or without surface wrinkles, in a controlled way.

## 2. Materials and Methods

### 2.1. Preparation of the Precursor Solution

All chemicals were obtained from Sigma-Aldrich, Germany, without any further purification; 0.25 M, 0.5 M and 1.0 M solutions of the precursor zinc acetate dehydrate were prepared in a mixture of ethyl alcohol and mono-ethanolamine (as a stabilizer). After that, PVA was added to each solution, which were then magnetically stirred and heated using a hotplate stirrer at 60 °C for 1 h to yield clear and homogeneous solutions, which were cooled to room temperature. Glass substrates were washed with a mild detergent followed by ultra-sonication in acetone, methanol and deionized water for 10 min; then, substrates were dried using nitrogen gas. Next, 4–5 droplets of the solution were spin coated onto the substrate using a spin coater (SPS Spin-150, Ingolstadt, Germany) at a specific speed for a particular time of less than 20 s.

### 2.2. Characterization of the Film

The crystallinity of ZnO films was examined using a Rigaku Ultima IV automated XRD (Cu target with λ = 0.15418 nm) in the 2θ range of 20–70 at a scan rate of 4° min^−1^ in thin film configuration. Elemental analysis was performed using a Thermo Fisher Scientific K-Alpha XPS with a pass energy of 50 eV at a base pressure of ~10^−9^ mbar. The morphology of the samples was examined using FEI quanta 250 FEG scanning electron microscopy. Samples’ topographies were examined using a Bruker Dimension Icon AFM in tapping mode. The film’s thickness was measured using an ellipsometer (M-88, J. A. Woollam, Lincoln, NE, USA) at 55°, 65° and 75° for each film.

## 3. Results and Discussion

Three different molarities of ZnO thin films were prepared using 0.25 M, 0.5 M and 1.0 M precursor solutions. All data that follow are for the 0.5 M solution, unless otherwise stated.

### 3.1. XRD Analysis

**Figure 1:**
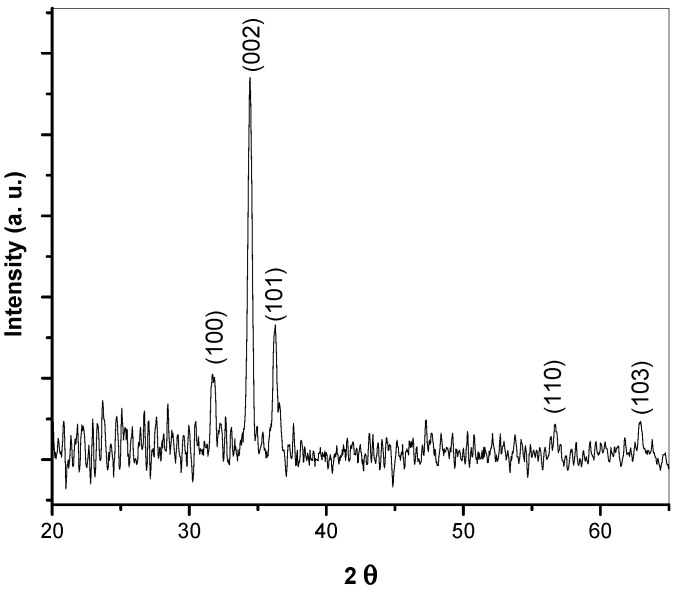
XRD pattern of annealed ZnO film.

XRD diffraction patterns of the annealed ZnO thin films with the diffraction angle 2θ varied between 20° and 70 are shown in Figure 1, where three ZnO film peaks, (100), (002) and (101), are evident. In addition, very weak ZnO peaks at (103) and (110) are in good agreement with JPCD no. 36-1451. This result may confirm that the film was polycrystalline with a wurtzite hexagonal structure. The intensity of the kinetically preferred (002) peak was the strongest among the peaks, which indicates preferred c-axis orientation. The average crystallite size was calculated according to Scherrer’s equation. Three main peaks were used after that; the average crystallite size was found to be 21.4 nm.

### 3.2. XPS Measurements

**Figure 2:**
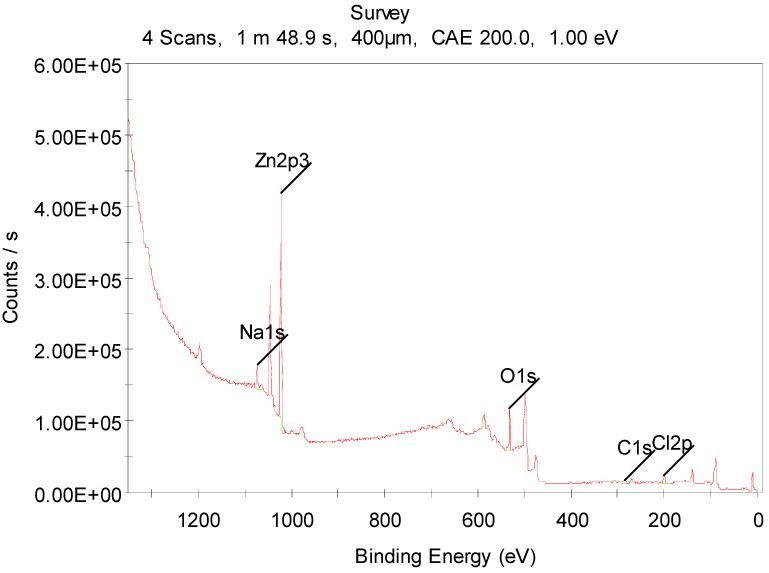
Overview spectrum of 0.5 M ZnO film with 1 eV resolution.

**Figure 3:**
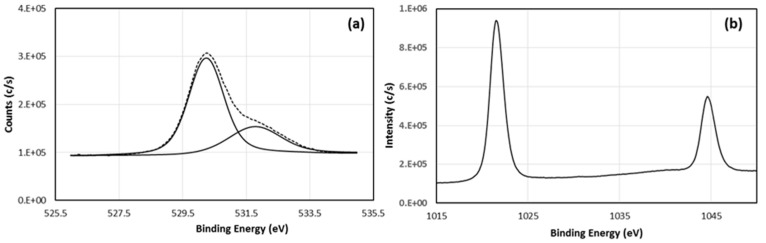
High resolution of XPS core level spectra of (**a**) O1s solid line: raw data, dotted line: fitting and (**b**) Zn2p.

XPS spectra were analyzed to investigate the film’s chemical composition. Before the film’s elemental analysis, it was etched using an argon ions beam to clean the surface of adsorbents (primarily CO_2_); where the CO_2_ peak disappears after the etching process is clearly visible in the survey spectrum. Survey spectra statistics show the Zn:O atomic ratio as 1.06:1. Figure 2 shows the overview spectrum of 0.5 M ZnO film with 1 eV resolution and Figure 3 shows oxygen and zinc spectra. A fine scan of the O1s spectrum, shown in Figure 3a, was deconvoluted into two sub-peaks at 530.28 and 531.78 eV with 70% and 30% ratios, respectively. The peak centered at 530.28 eV was attributed to oxygen at the oxide lattice and the peak centered at 531.78 eV was attributed to Zn(OH)_2_ [40]. Figure 3b shows a fine scan of the zinc XPS spectrum, which indicates that the zinc was in an oxidation state; symmetrical peaks Zn2p3/2 and Zn2p1/2 at 1021.6 eV and 1044.6 eV, respectively, indicate the oxidation state of zinc referenced to adventitious C1s peak at 284.8 eV.

### 3.3. SEM Analysis

To examine wrinkle formation, two film samples we re coated under the same conditions: 2500 rpm for 40 s using 0.5 M solution. One sample was dried at room temperature for one day; the other was dried in an oven at 200 °C for 1 h at a heating rate of 20 °C/min and then cooled in the oven after it was switched off. These drying modes were selected to examine the formation of wrinkles.

**Figure 4:**
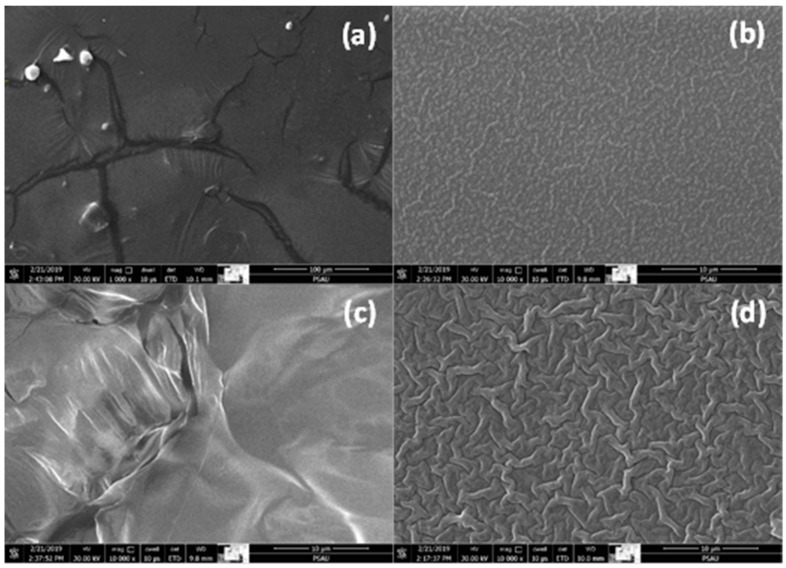
SEM images of (**a**,**b**) 0.5 M RT film, dried and thermally dried, respectively, and (**c**,**d**) 1.0 M RT film, dried and thermally dried, respectively.

Figure 4 shows SEM images for films dried using both methods at different magnifications. Figure 3a,c show air-dried films and Figure 3b,d show oven-dried films at different magnifications. No wrinkles were observed in air-dried films, whereas wrinkles were observed in oven-dried films. This observation confirms that thermal contraction of the substrate causes these wrinkles. During the substrate’s thermal expansion, the film widened and thinned to the appropriate thickness and density without thermal stress. After the film dried, it became denser, tightened and compression stress started to build, which resulted in wrinkle formation. This behavior is attributed to the substrate’s cooling, which contracted the film’s constituents.

**Figure 5:**
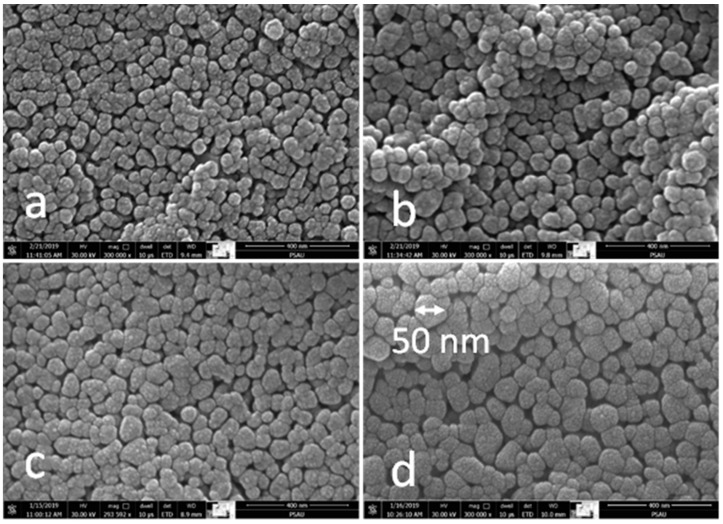
SEM images of (**a**,**b**) 0.5 M film, with and without wrinkles, and (**c**,**d**) 1 M film, with and without wrinkles.

The particle size of all obtained films was less than 50 nm; this can be seen in Figure 5a,b, which show 0.5 M film with and without wrinkles, and Figure 5c,d, which show the same for 1 M film.

**Figure 6:**
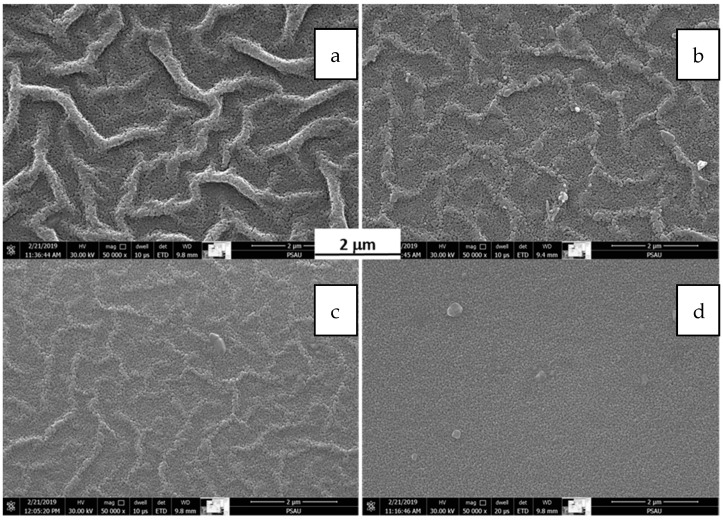
SEM images of ZnO films coated at different spinning speeds: (**a**) 2000 rpm, (**b**) 2500 rpm, (**c**) 3000 rpm and (**d**) 3500 rpm.

Figure 6 shows SEM images produced using the ETD detector (secondary electrons mode) of films prepared in the same conditions with the exception of spin coating. For these films, 0.5 M solution was used in the spin coating process for 40 s at speeds of 2000, 2500, 3000 and 3500 rpm, respectively. The average size of ZnO particles was ~50 nm; sizes slightly decreased as spin coating speed increased, which is in good agreement with previous research indicating that particle size ranged from 250 nm to 311 nm [41]. The images illustrate that the development of surface structure produced using reduced spin coating speed elucidated as surface wrinkles. At a low spin coating speed, the wrinkles’ presence was significant, as shown in Figure 6a. As the spinning speeds increased the wrinkles’ heights decreased, as shown in Figure 6b,c, and eventually vanished at 3500 rpm, as shown in Figure 6d. All films processed using the 1 M solution had wrinkled surfaces at all speeds up to 4000 rpm; at this speed the solution partially flew off the substrate. In contrast, all films processed using the 0.25 M solution show flat surfaces at all speeds up to 4000 rpm, with no sign of wrinkling.

**Figure 7:**
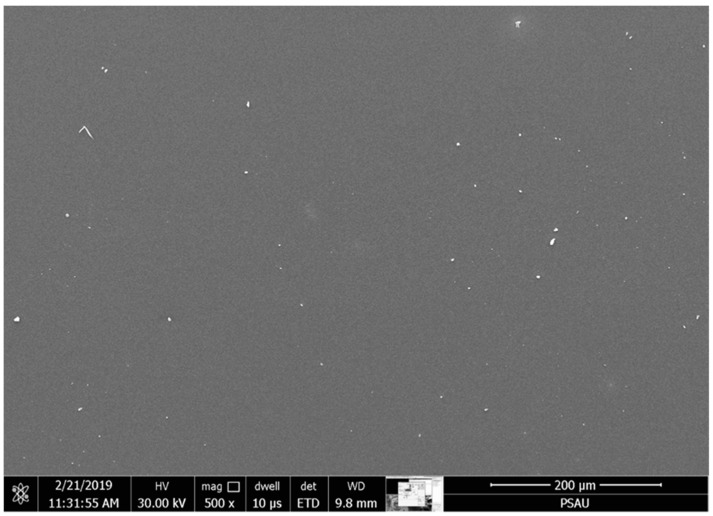
SEM image of ZnO film coated at 3500 rpm.

Using PVA as a binding polymer helped produce a uniform film over the whole substrate area (whether or not it was wrinkly). Figure 7 shows an SEM image for one of the films sized ~1 × 1 mm. The film was consistently distributed and free of any defects; white dots that appeared may be attributed to work that was not performed in a cleanroom. It is well known that films coated with gold using thermal sputtering provide high-resolution SEM images. Additionally, gold coating consists of 10 nm particles, which appear as white dots in these images. At a higher spinning speed, e.g., at 4000 rpm, the films suffered from depleted areas where the film flew off the substrate.

### 3.4. AFM Measurements

**Figure 8:**
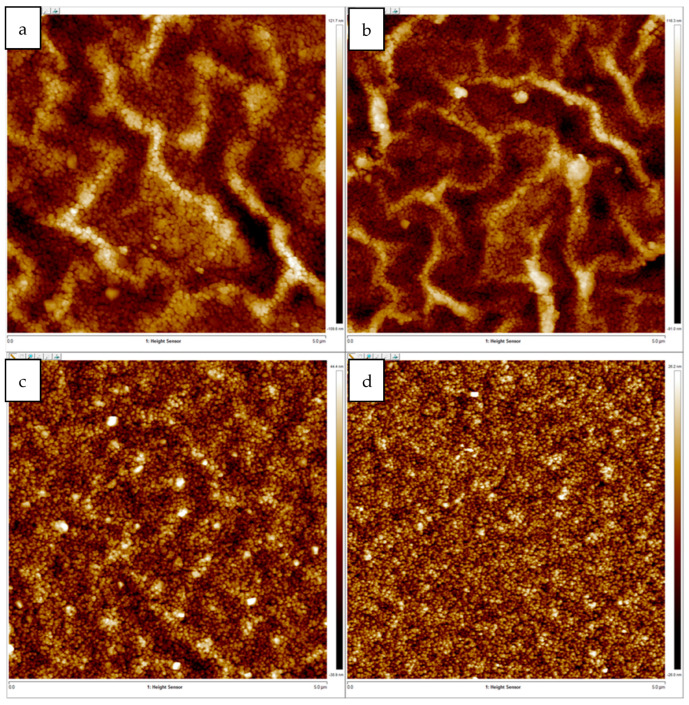
(**a**–**d**) AFM images for the same films previous used for SEM imaging.

Surface roughness and wrinkles’ heights were studied using AFM in tapping mode. Figure 8 shows AFM images for the same films previous used for SEM imaging. Surfaces’ root mean square roughness, R_q_, were 26.3, 23.5, 9.0 and 6.2 nm for film spun at 2000, 2500, 3000 and 3500 rpm, respectively.

**Figure 9:**
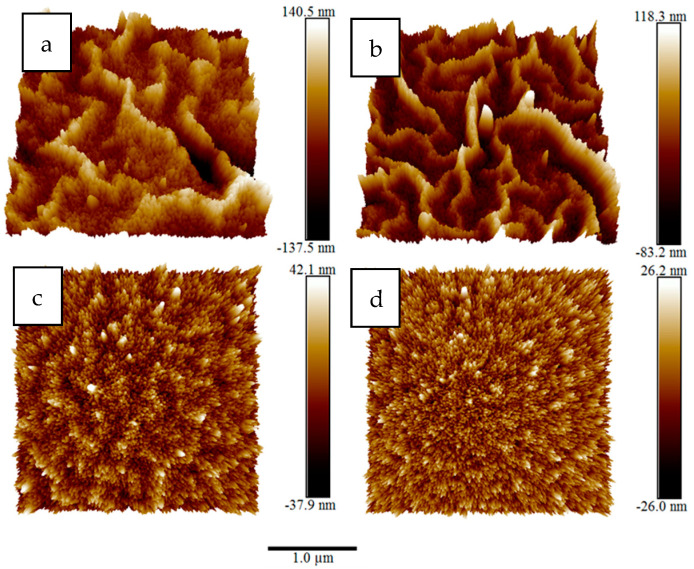
Three-dimensional AFM images of ZnO films coated at different spinning speeds: (**a**) 2000 rpm, (**b**) 2500 rpm, (**c**) 3000 rpm and (**d**) 3500 rpm.

Figure 9 shows 3D AFM images of the same films. The 3D AFM images clearly show the reduction in surface roughness and wrinkles’ heights as spinning speed increased.

**Figure 10:**
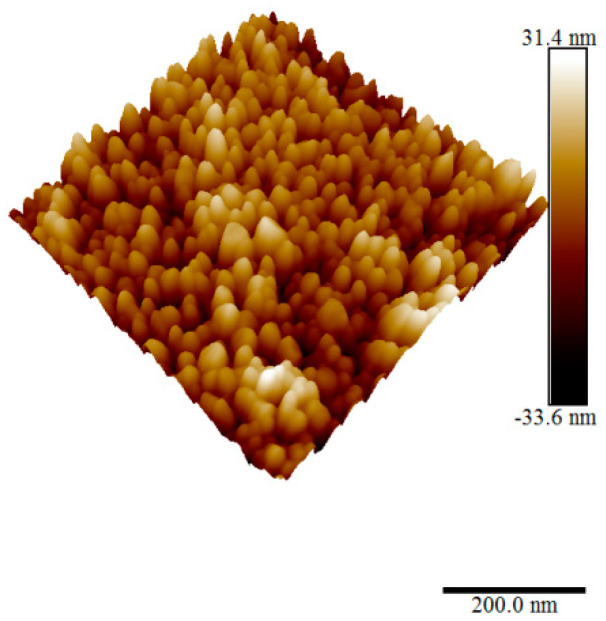
Three-dimensional AFM image of nanostructured ZnO film.

Figure 10 shows a high-resolution 3D AFM image of one sample showing the nanostructure nature of the film.

### 3.5. Ellipsometery Measurements

**Figure 11:**
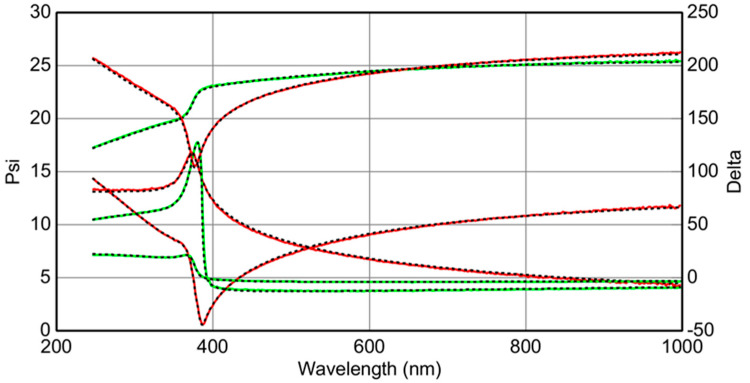
Fitting of measured data for ellipsometric parameters Ψ and Δ for sample spinning speed of 3500 rpm using variable angle spectroscopic ellipsometric (VASE) data where the red line is the measured and the green line is the fitted.

The thicknesses of films shown in Figure 5 were measured using spectroscopic ellipsometry (SE) by adjusting the fitting parameters to generate the same experimental results, which include film thickness. Figure 11 shows an example of SE data fitting for film spun at 3500 rpm. Matte-finish adhesive tape was affixed to the substrate’s back surface to scatter back surface reflections and fit SE data. To obtain the exact thickness of the film; we tuned the film parameters and introduced the substrate parameter to generate the same experimental data.(thanks to Fabry Perot interferometry). SE data fitting for film spun at 3500 rpm showed that the films’ thicknesses were 83.0, 78.5, 61.3 and 56.3 nm for Figure 5a–d, respectively. Comparing AFM and SE data revealed the conditions required to produce wrinkled free film as follows: ~50 nm ZnO nanoparticle size, 56.3 nm film thickness and 6.2 nm roughness. From this, it can be concluded that the conditions for obtaining wrinkle free film include at most two stacked layers of ZnO nanoparticles; a thick film can be obtained after drying the first layer and applying a second coat before the annealing process.

Adding binding PVA polymer to the precursor solution helped produce uniform films on an extended area and eliminated compression stress (which can cause films to develop extra wrinkles) during the cooling process after film annealing. One film was exposed to thermal expansion during the drying process at a higher temperature, whereas the second film was dried at ambient temperature. Drying at a higher temperature led to mechanical stress between film particles on the first film as the substrate cooled. Adjusting the procurer solution contents of the zinc acetate and using the appropriate spinning speed formed two stacked layers of film with a smooth surface free of wrinkling. Reducing the spinning speed and increasing the molarity of zinc acetate led to wrinkle formationmoreover, it could produce ZnO films with tunable wrinkle heights.

## 4. Conclusions

In summary, ZnO thin film was prepared using a timesaving method in less than one hour, employing PVA polymer as a binder solution to control the precursor’s viscosity instead of using a time-consuming aging technique. Moreover, the suggested method achieved film uniformity, which extended through the entire substrate. The primary factors which affect and control the film’s surface morphology, such as precursor molarity, PVA binder content and spin coating speed, were studied and led to significant findings:

1. For the 1.0 molar solution, the film surface had a wrinkled structure regardless of the spin coating speed.
2. For the 0.5 molar solution, it was possible to tune the film surface morphology between wrinkle-free and wrinkled by varying the spin coating speed.
3. The film had a critical thickness after which it wrinkled regardless of the spin coating speed.

The obtained film can be implemented as a buffer layer (electron selective layer, ESL) in polymer or perovskite solar cells, and employed to study the effectiveness of ESL surface morphology (in particular the film’s wrinkled surface) on solar cells’ performance.

## Data Availability

Not applicable.
